# Supplementary material for: Runx2 is essential for the transdifferentiation of chondrocytes into osteoblasts
Source: PLoS Genet. 2020 Nov 30;16(11):e1009169. doi: 10.1371/journal.pgen.1009169 (PMC7728394; doi:10.1371/journal.pgen.1009169)
Supplement: S1 Fig — (A) Targeting vector and strategy for generating a Runx2-flox mouse line. (B) Southern blot analyses of genomic DNA from wild-type and targeted ES cells digested with XbaI or SacI using 5’ or 3’ probe shown in (A), respectively. (C) PCR for genotyping to detect wild-type and floxed alleles in mice using F and R primers. (PDF) [file pgen.1009169.s001.pdf]

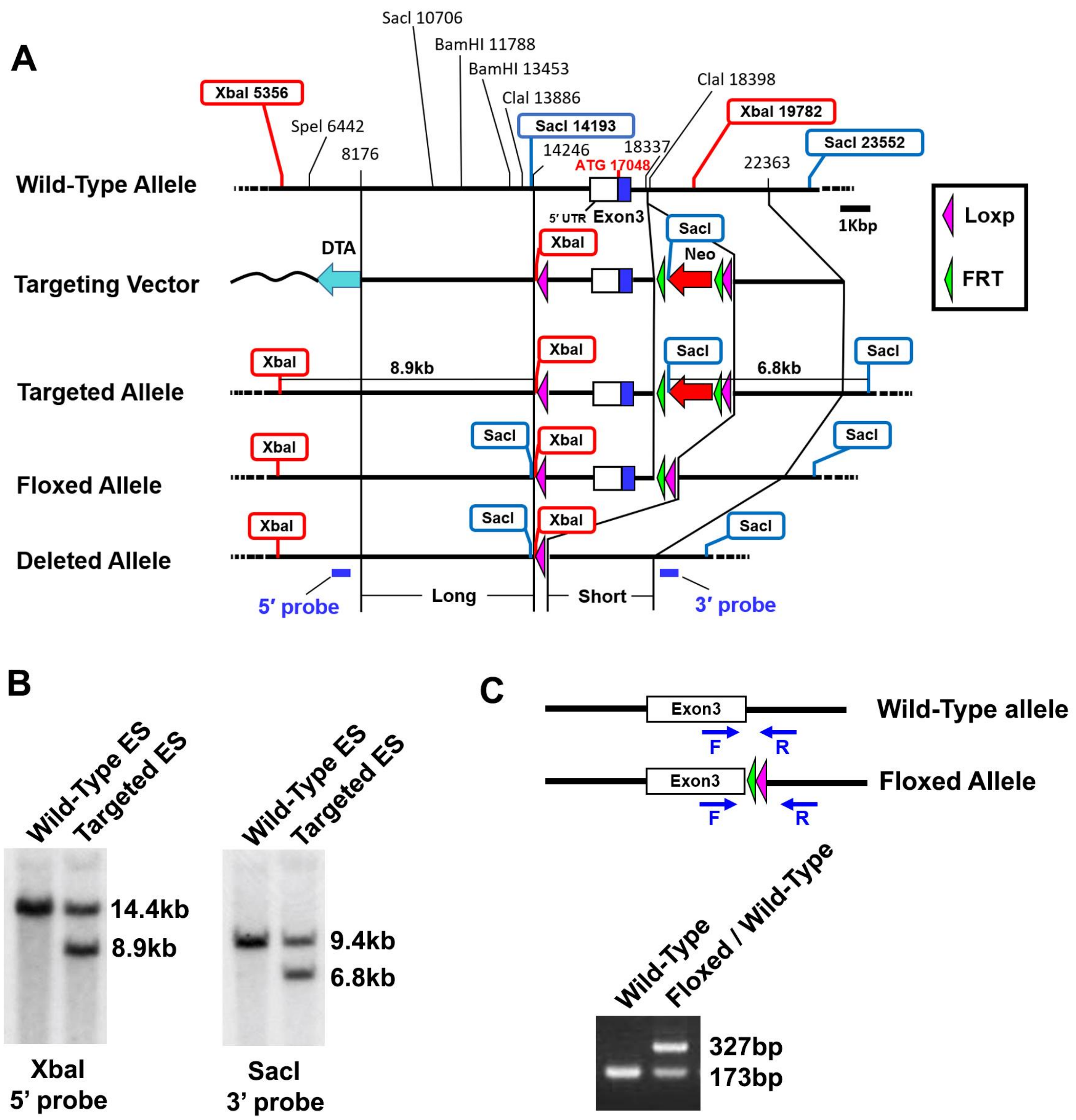

S1 Fig

Generation of *Runx2* flox mice

(A) Targeting vector and strategy for generating a *Runx2*-flox mouse line. (B) Southern blot analyses of genomic DNA from wild-type and targeted ES cells digested with *XbaI* or *SacI* using 5' or 3' probe shown in (A), respectively. (C) PCR for genotyping to detect wild-type and floxed alleles in mice using F and R primers.
