# Supplementary material for: Runx2 is essential for the transdifferentiation of chondrocytes into osteoblasts
Source: PLoS Genet. 2020 Nov 30;16(11):e1009169. doi: 10.1371/journal.pgen.1009169 (PMC7728394; doi:10.1371/journal.pgen.1009169)
Supplement: S2 Fig — Immunohistochemistry using anti-Hmgb1 antibody was performed in femoral sections of Runx2fl/fl (A, C, E, G) and Runx2fl/fl Cre (B, D, F, H) mice at E16.5. (A-D) Staining with DAPI. (E-H) Immunohistochemical analysis using anti-Hmgb1 antibody. The boxed regions in A, B, E, F were magnified in C, D, G, H, respectively. The dotted lines in E and F show the border between the growth plate and bone marrow. The boxed cells in G and H were magnified in the windows. Scale bars: 50 μm (A, B, E, F), 20 μm (C, D, G, H). (I) Cell area/total area, DyLight+ area, and intensity of DyLight in the boxed regions without bone marrow area in E and H (hypertrophic and terminal hypertrophic chondrocyte layers) were compared between Runx2fl/fl and Runx2fl/fl Cre mice. The number of mice analyzed: Runx2fl/fl: 4, Runx2fl/fl Cre: 4. Monoclonal rabbit anti-Hmgb1 antibody (Cell Signaling, Danvers, MA) and goat anti-rabbit IgG H&L (DyLight 488) secondary antibody (Abcam, Cambridge, UK) were used for the immunohistochemical analysis. (PDF) [file pgen.1009169.s002.pdf]

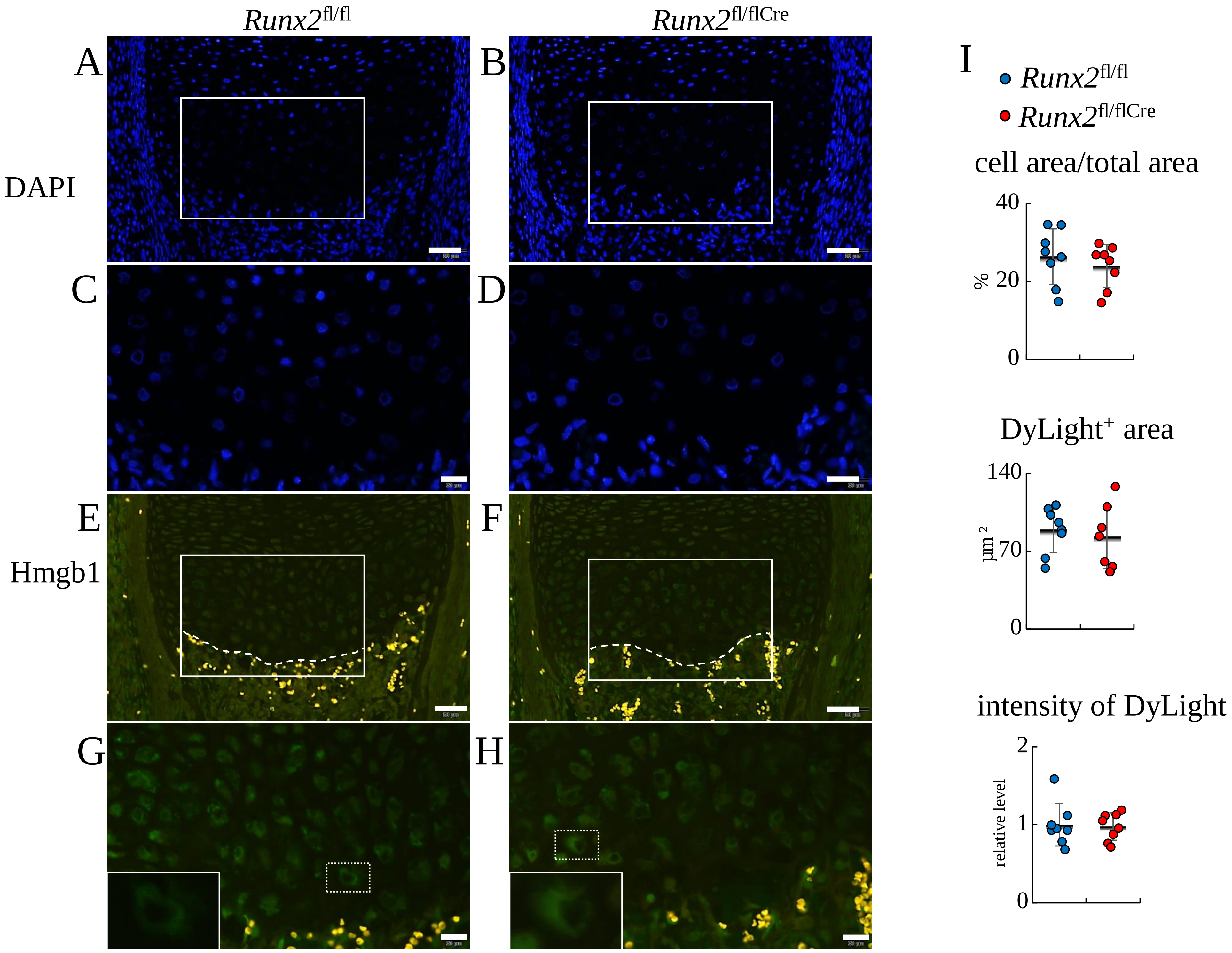

S2 Fig

Immunohistochemistry of Hmgb1

Immunohistochemistry using anti-Hmgb1 antibody was performed in femoral sections of *Runx2*$^{fl/fl}$ (A, C, E, G) and *Runx2*$^{fl/fl\ Cre}$ (B, D, F, H) mice at E16.5. (A-D) Staining with DAPI. (E-H) Immunohistochemical analysis using anti-Hmgb1 antibody. The boxed regions in A, B, E, F were magnified in C, D, G, H, respectively. The dotted lines in E and F show the border between the growth plate and bone marrow. The boxed cells in G and H were magnified in the windows. Scale bars: 50 μm (A, B, E, F), 20 μm (C, D, G, H). (I) Cell area/total area, DyLight^{+} area, and intensity of DyLight in the boxed regions without bone marrow area in E and H (hypertrophic and terminal hypertrophic chondrocyte layers) were compared between *Runx2*$^{fl/fl}$ and *Runx2*$^{fl/fl\ Cre}$ mice. The number of mice analyzed: *Runx2*$^{fl/fl}$: 4, *Runx2*$^{fl/fl\ Cre}$: 4. Monoclonal rabbit anti-Hmgb1 antibody (Cell Signaling, Danvers, MA) and goat anti-rabbit IgG H&L (DyLight 488) secondary antibody (Abcam, Cambridge, UK) were used for the immunohistochemical analysis.
