# Supplementary material for: Runx2 is essential for the transdifferentiation of chondrocytes into osteoblasts
Source: PLoS Genet. 2020 Nov 30;16(11):e1009169. doi: 10.1371/journal.pgen.1009169 (PMC7728394; doi:10.1371/journal.pgen.1009169)
Supplement: S3 Fig — (A-C) Negative control for β-galactosidase staining using femoral sections from Runx2fl/fl mice at E15.5 (A), Runx2fl/+ mice at E16.5 (B), and Runx2fl/+ LacZ mice at E17.5 (C). The boxed regions in A-C and A’-C’ were magnified in A’-C’ and A”-C”, respectively. (D and E) Immunohistochemical analysis of femoral sections using anti-Col1a1 antibody in Runx2fl/fl (D) and Runx2fl/flCre (E) embryos at E15.5. Scale bars: 100 μm (A–C’, D, E), 50 μm (A”–C”). Two mice for each genotype were analyzed. (PDF) [file pgen.1009169.s003.pdf]

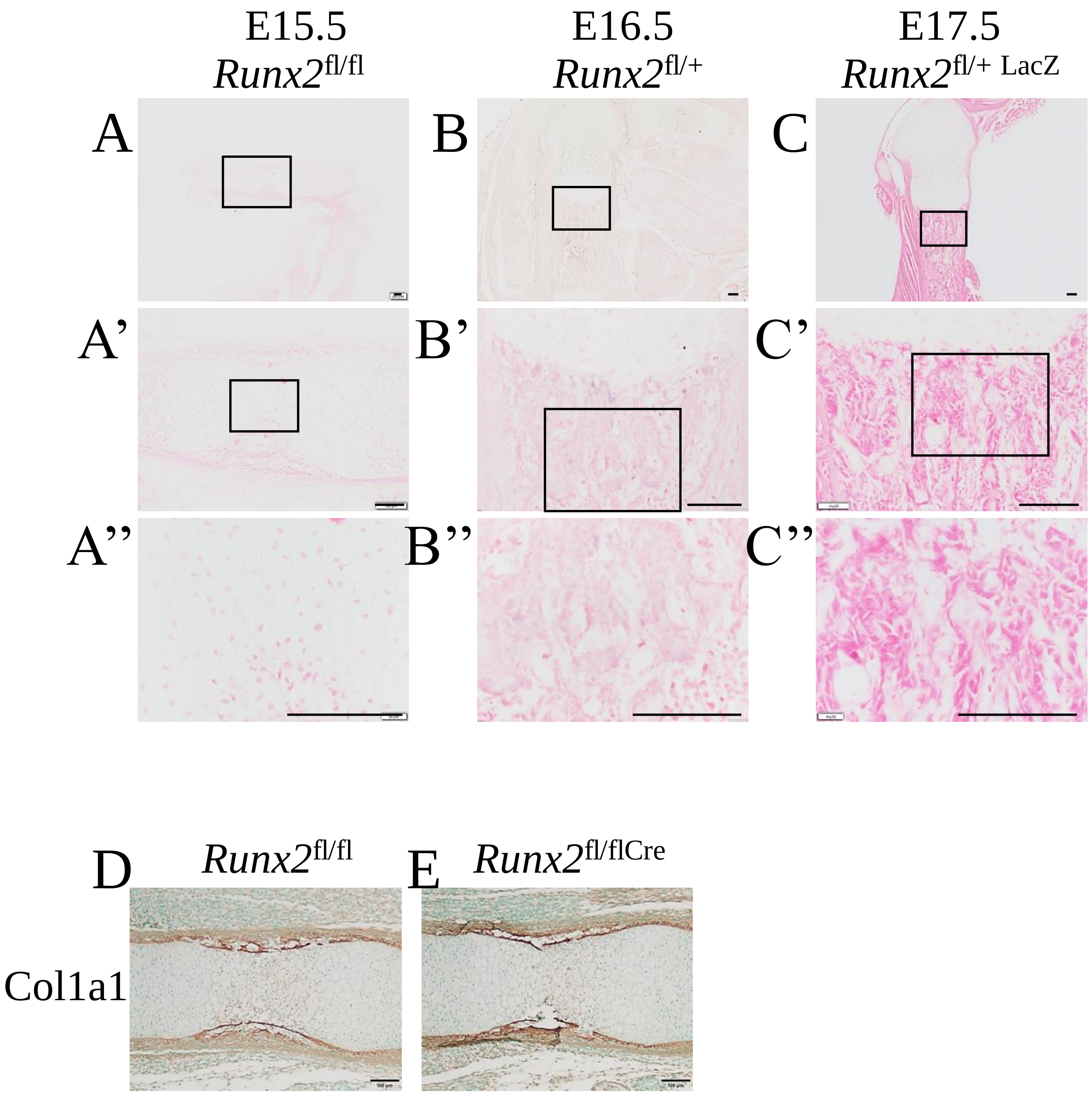

S3 Fig

β-galactosidase staining and immunohistochemistry of Col1a1

(A-C) Negative control for β-galactosidase staining using femoral sections from *Runx2*$^{fl/fl}$ mice at E15.5 (A), *Runx2*$^{fl/+}$ mice at E16.5 (B), and *Runx2*$^{fl/+\ LacZ}$ mice at E17.5 (C). The boxed regions in A-C and A'-C' were magnified in A'-C' and A''-C'', respectively. (D and E) Immunohistochemical analysis of femoral sections using anti-Col1a1 antibody in *Runx2*$^{fl/fl}$ (D) and *Runx2*$^{fl/flCre}$ (E) embryos at E15.5. Scale bars: 100 μm (A–C', D, E), 50 μm (A''–C''). Two mice for each genotype were analyzed.
