# Supplementary material for: Runx2 is essential for the transdifferentiation of chondrocytes into osteoblasts
Source: PLoS Genet. 2020 Nov 30;16(11):e1009169. doi: 10.1371/journal.pgen.1009169 (PMC7728394; doi:10.1371/journal.pgen.1009169)
Supplement: S4 Fig — (A) Construct of the transgene. * Intron from SV40 containing splice donor and acceptor sites. ** polyadenylation signal from SV40. (B) Whole embryo at E14.5. (C-E) Frozen section of femur at P2. The boxed regions in C were magnified in D and E. Pictures were taken using fluorescence microscope (BZ-X710, KEYENCE, Osaka Japan). Scale bars: 1 mm (B), 500 μm (C), 100 μm (D, E). (PDF) [file pgen.1009169.s004.pdf]

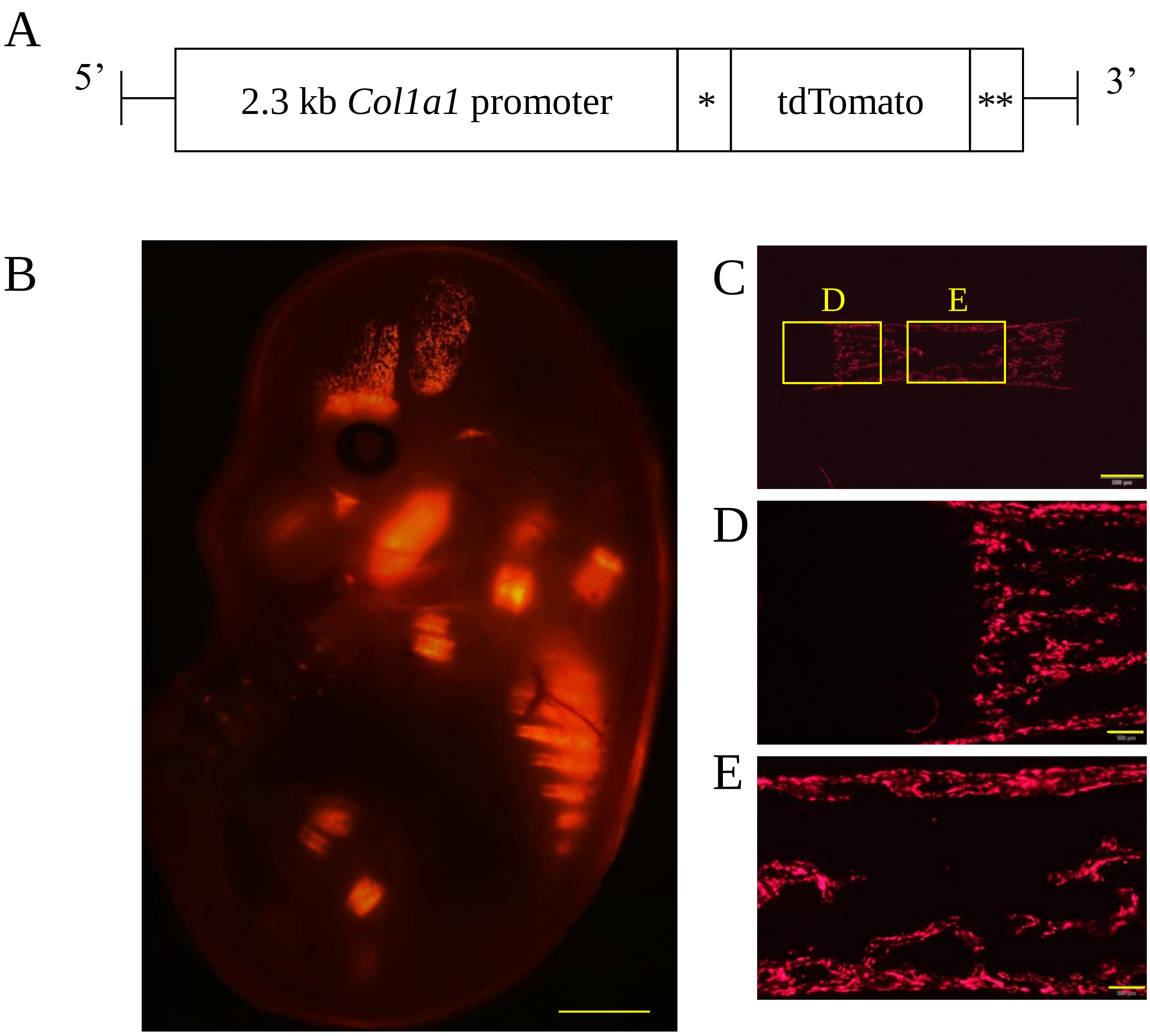

S4 Fig

Generation of 2.3 kb *Col1a1* promoter tdTomato transgenic mice

(A) Construct of the transgene. * Intron from SV40 containing splice donor and acceptor sites. ** polyadenylation signal from SV40. (B) Whole embryo at E14.5. (C-E) Frozen section of femur at P2. The boxed regions in C were magnified in D and E. Pictures were taken using fluorescence microscope (BZ-X710, KEYENCE, Osaka Japan). Scale bars: 1 mm (B), 500 µm (C), 100 µm (D, E).
