# Supplementary material for: Runx2 is essential for the transdifferentiation of chondrocytes into osteoblasts
Source: PLoS Genet. 2020 Nov 30;16(11):e1009169. doi: 10.1371/journal.pgen.1009169 (PMC7728394; doi:10.1371/journal.pgen.1009169)
Supplement: S5 Fig — (A-C) β-galactosidase staining of femoral sections from Runx2fl/fl (A), Runx2fl/+Cre LacZ (B), and Runx2fl/flCre LacZ (C) mice at 3 weeks of age. The boxed regions in A-C and A’-C’ were magnified in A’-C’ and A”-C”, respectively. The secondary ossification center is shown. Arrows in B” indicate osteoblastic cells. (D-G) Micro-CT images of femurs at 6 and 20 weeks of age. The trabecular bone between the two lines with the distance of 0.36 mm in the secondary ossification center was analyzed in Figs 10M and 11M. Scale bars: 200 μm (A-C), 20 μm (A’-C’), 10 μm (A”-C”), 0.5 mm (D-G). (PDF) [file pgen.1009169.s005.pdf]

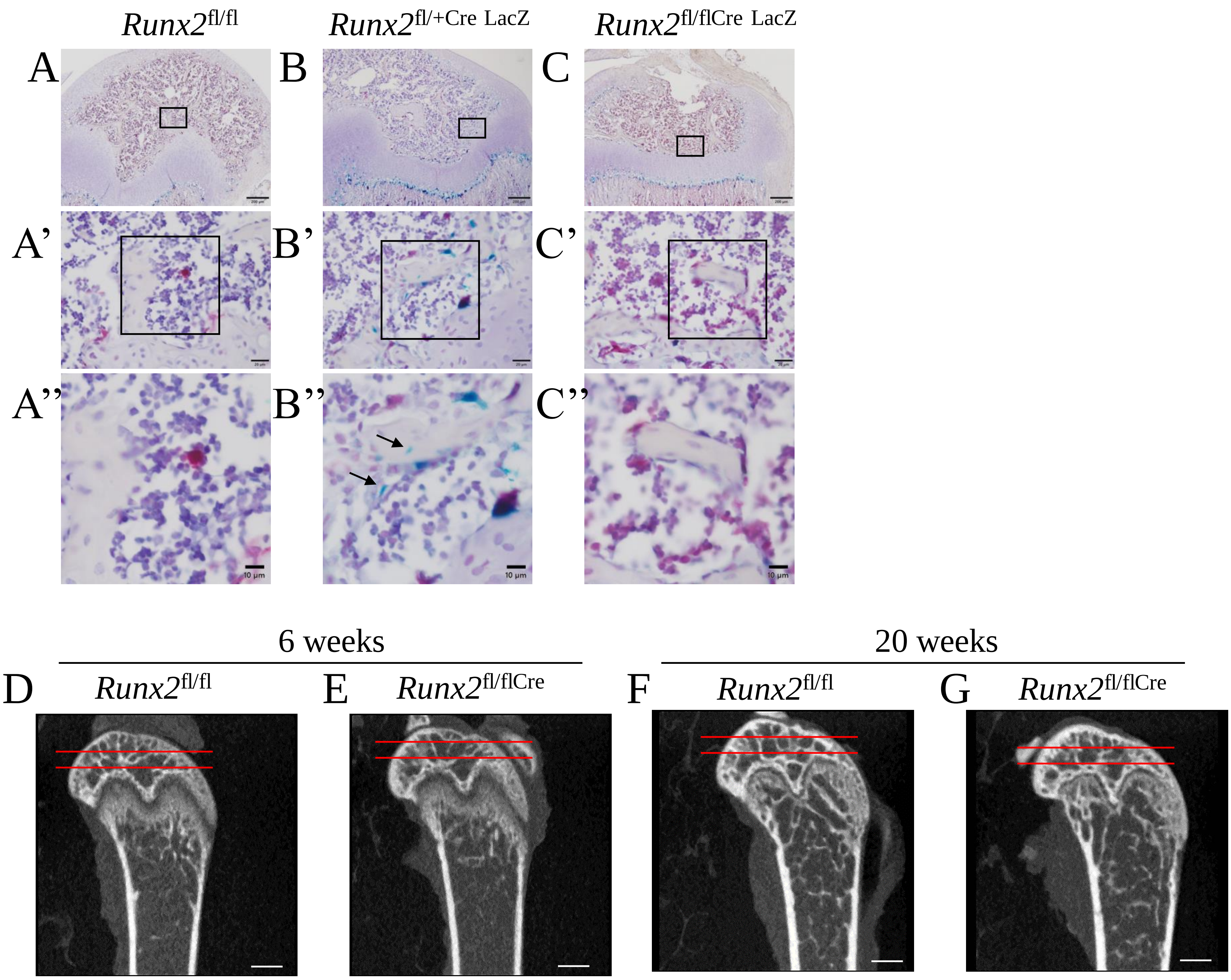

S5 Fig

β-galactosidase staining and micro-CT analysis

(A-C) β-galactosidase staining of femoral sections from *Runx2*$^{fl/fl}$ (A), *Runx2*$^{fl/+Cre\ LacZ}$ (B), and *Runx2*$^{fl/flCre\ LacZ}$ (C) mice at 3 weeks of age. The boxed regions in A-C and A’-C’ were magnified in A’-C’ and A”-C”, respectively. The secondary ossification center is shown. Arrows in B” indicate osteoblastic cells. (D-G) Micro-CT images of femurs at 6 and 20 weeks of age. The trabecular bone between the two lines with the distance of 0.36 mm in the secondary ossification center was analyzed in Figs. 10M and 11M. Scale bars: 200 μm (A-C), 20 μm (A’-C’), 10 μm (A”-C”), 0.5 mm (D-G).
