# Supplementary material for: Runx2 is essential for the transdifferentiation of chondrocytes into osteoblasts
Source: PLoS Genet. 2020 Nov 30;16(11):e1009169. doi: 10.1371/journal.pgen.1009169 (PMC7728394; doi:10.1371/journal.pgen.1009169)
Supplement: S1 Table — (PDF) [file pgen.1009169.s006.pdf]

S1 Table Primer sequences for real-time RT-PCR

	Forward	Reverse
	5'---------------------3'	5'---------------------3'
Actb	CCACCCGCGAGCACAGCTTC	TTGTCGACGACCAGCGCAGC
Runx2	AACAAGACCCTGCCCGTG	TGAAACTCTTGCCTCGTCCG
Col10a1	ATATGCTGCCTCAAATACCC	CTCTTATGGCGTATGGGAT
Mmp13	CTTCTGGCACACGCTTTTCC	ATGGGAAACATCAGGGCTCC
Ibsp	TGGAGACGGCGATAGTTC	CTAGCTGTTACACCCGAGAG
Spp1	GCAGAATCTCCTTGCGCCAC	CGAGTCCACAGAATCCTCGC
Vegfa	GGAGATCCTTCGAGGAGCACTT	GGCGATTTAGCAGCAGATATAAGAA
Tnfsf11	CAAGCTCCGAGCTGGTGAAG	CCTGAACTTTGAAAGCCCCA
Tnfsl11b	AAGAGCAAACCTTCCAGCTGC	CACGCTGCTTTCACAGAGGTC
Csf1	GGATGATCCTGTTTGCTACC	GAGTCTCATGGAAAGTTCGG
Bcl2	GTGGCCTTCTTTGAGTTCG	TTCAGAGACAGCCAGGAGA
Bcl2l1	GCAGGTATTGGTGAGTCG	GGCTGCTGCATTGTTCCC
Pten	GGAAAGGGACGGACTGGTGT	GACTGGGAATTGTGACTCCC
Bcl2l11	CTCCCTACAGACAGAACCGC	CGTTGAACTCGTCTCCGATC
Fas	CCCTGACCCAGAATACCAAG	CAAGGGTTCCATGTTCACAC
Bad	GACCAGCAGCCCAGAGTATG	CCTGGCTGGTCCTCAGTGAG
Pmaip1	GTACACGCCGACCGTAATGG	GGAGGAATTCTCGGCTGCAG
Bax	GTGAGCGGCTGCTTGTCTGG	CTTCCAGATGGTGAGCGAGG
Bnip3	GGCGAGAAAAACAGCACTCT	GGGGGAATATTTTCTGGTCG
