# Supplementary material for: Runx2 is essential for the transdifferentiation of chondrocytes into osteoblasts
Source: PLoS Genet. 2020 Nov 30;16(11):e1009169. doi: 10.1371/journal.pgen.1009169 (PMC7728394; doi:10.1371/journal.pgen.1009169)
Supplement: Attachment — Submitted filename: Responses to the reviewer.docx [file pgen.1009169.s007.docx]

Responses to the reviewer's comments and questions

Reviewer #1: This paper explores the functions of Runx2 in hypertrophic chondrocytes through conditional inactivation with Col10a1-Cre. This work builds on previous work showing that Runx2 is necessary for chondrogenesis and osteogenesis. In particular Runx2 ko mice only form cartilage, the chondrocytes fail to differentiate into hypertrophic chondrocytes and vascular invasion of the cartilage anlage does not occur. Interestingly in this work, with inactivation of Runx2 in hypertrophic chondrocytes, vascular invasion still occurs, hypertrophy still occurs, but hypertrophic chondrocytes do not differentiate into trabecular osteoblasts, and apoptosis hypertrophic chondrocytes was increased. However, as mice age, the bone phenotypes normalized and no differences between controls and knockouts were observed in adult mice.

Overall, the study is rigorously performed and the conclusions are consistent with the data. The overall knowledge gained from this study is somewhat incremental, in that the main conclusion is that Runx2 is necessary for osteoblast differentiation.

Major Comments
In figure 2, markers of the primary spongiosa, Mmp13, Ibsp, and Spp1, are reduced or absent at E16.5. Is this a delay in formation of the primary spongiosa or does it never form? Analysis of later time points with these markers should address this.

The reduction of *Mmp13*, *Ibsp*, and *Spp1* expression at E16.5 was due to their reduced expression in the terminal hypertrophic chondrocytes and a delay in the formation of primary spongiosa.

First, we compared the expression of the genes in hypertrophic and terminal hypertrophic layers between *Runx2*^fl/fl^ and *Runx2*^fl/flCre^ mice at E15.5 using laser capture microdissection system (Fig. 1Q, R). *Mmp13*, *Ibsp*, and *Spp1* expression in the terminal hypertrophic chondrocytes was reduced in *Runx2*^fl/flCre^ mice compared with that in *Runx2*^fl/fl^ mice. *Mmp13* was strongly expressed in the terminal hypertrophic chondrocytes but not in osteoblasts, and *Ibsp* and *Spp1* were strongly expressed in both terminal hypertrophic chondrocytes and osteoblasts (Fig. 2J, K, M, N, P, Q). *Mmp13*, *Ibsp*, and *Spp1* expression was severely reduced in *Runx2*^fl/flCre^ mice at E16.5 (Fig. 2J-R, V), but the reduction of *Ibsp* and *Spp1* expression was mild or marginal at E17.5 due to the gradually progressed primary spongiosa formation (Fig. 8E-H, N, O).

It is shown that Vegfa is reduced in the primary spongiosa but that vascular invasion is normal. However, it is not clear if there is still a delay in the vascular invasion in the Runx2 conditional KO mice. The normal vascular invasion should be demonstrated with an immunostain for endothelial cells.

We performed immunohistochemistry using anti-CD34 antibody to detect the endothelial cells (Fig. 3I-K). CD34-positive endothelial cells were similarly detected in *Runx2*^fl/fl^ and *Runx2*^fl/flCre^ mice at E16.5, showing that the vascular invasion into the cartilage is not impaired in *Runx2*^fl/flCre^ mice.

How does the reduced expression of Vegfa reported here compare with the phenotype of chondrocyte-specific inactivation of Vegfa?

Vegfa conditional knockout mice using *Col2a1*-Cre, which directs the transgene expression to chondrocytes and perichondral cells, showed delayed vascular invasion into the cartilage (Zelzer et al, 2004). The delayed vascular invasion is due to the lack of Vegfa expression in terminal hypertrophic chondrocytes and perichondrial osteoblasts, both of which strongly express Vegfa. Similarly, Vegfa conditional knockout mice using *Sp7*-Cre, which directs the transgene expression to prehypertrophic chondrocytes and preosteoblasts in the perichondrium, showed delayed vascular invasion into the cartilage (Duan et al., 2015). The delayed vascular invasion is also due to the lack of Vegfa expression in terminal hypertrophic chondrocytes and perichondrial osteoblasts. Vegfa expression in the terminal hypertrophic chondrocytes was reduced in *Runx2*^–/–^ mice and *Runx2*^fl/flCre^ mice, but vascular invasion into the cartilage never occurs in *Runx2*^–/–^ mice but normally occurred in *Runx2*^fl/flCre^ mice. The difference of the two mouse models is the presence of perichondrial osteoblasts in *Runx2*^fl/flCre^ mice but their absence in *Runx2*^–/–^ mice. Thus, these findings indicate that Vegfa expression in perichondrial osteoblasts is sufficient for the vascular invasion into the cartilage. These discussion was described in the Discussion section (pages 23-24).

The mechanisms that could explain the increased osteoclasts in Runx2 conditional KO mice should be investigated by examining expression of RANKL, OPG and GM-CSF compared to expression of DAMPs.

We examined the expression of *Tnfsf11* (Rankl), *Tnfrsf11b* (Opg), and *Csf1* (m-csf) in the hypertrophic and terminal hypertrophic layers by laser capture microdissection at E15.5, when the vascular invasion is going to start (Fig. 1Q, R). The expression of *Tnfsf11* and *Csf1* in *Runx2*^fl/flCre^ mice was lower than that in *Runx2*^fl/fl^ mice, and *Tnfrsf11b* expression was similar between *Runx2*^fl/fl^ and *Runx2*^fl/flCre^ mice. It is consistent with the previous finding that Runx2 positively regulates *Tnfsf11* expression (Enomoto et al., 2003). We also extracted RNA from diaphyses of femurs and tibiae at E16.5, which contained hypertrophic and terminal hypertrophic chondrocytes, osteoblasts, and hematopoietic cells. *Tnfsf11* expression was similar between *Runx2*^fl/fl^ and *Runx2*^fl/flCre^ mice (Fig. 3Y). These findings suggest that *Tnfsf11* expression in osteoblasts was upregulated and osteoclastogenesis was enhanced by *Tnfsf11* on the osteoblasts but not by *Tnfsf11* on the terminal hypertrophic chondrocytes in *Runx2*^fl/flCre^ mice.

High mobility group box 1 (Hmgb1) is one of the DAMPs, which are released from necrotic cells and induce *Tnfsf11* expression in osteoblast lineage cells. Hmgb1 is normally localized in the nucleus and translocated to the cytosol with many types of cellular stress (Gorgulho et al, 2019). Thus, we examined the localization of Hmgb1 protein. Unexpectedly, Hmgb1 protein was dominantly localized in cytosol in the hypertrophic and terminal hypertrophic chondrocytes in both *Runx2*^fl/fl^ and *Runx2*^fl/flCre^ mice, and the stained area and intensity were also similar between them (Supplementary Figure 2). Thus, we could not detect the increased Hmgb1 translocation to cytosol in *Runx2*^fl/flCre^ mice. As DAMPs exhibit their effect on *Tnfsf11* induction after necrosis, we should detect the DAMPs released from necrotic cells. However, we could not find the method to detect the released DAMPs in vivo.

These results were described in the Result section (pages 13-14).

The failure of transdifferentiation should be assessed by co-staining for the lineage marker, bGal and an osteoblast markers and quantifying the ratio of lineage marked osteoblasts to total osteoblasts in the primary spongiosa in embryonic and mature bone. An antibody to bGal should exclude staining of osteoclasts. Alternatively, a fluorescent lineage reporter could be used instead of LacZ. The literature suggests that transdifferentiation only accounts for about 20% of osteoblasts in the primary spongiosa. This should be rigorously evaluated here.

To more precisely evaluate the transdifferentiation of chondrocytes into osteoblasts, we mated *Runx2*^fl/flCre^ mice with Rosa26-CAG-loxP-mTFP1 (Rosa) reporter mice and 2.3 kb *Cola1* promoter-tdTomato (tomato) mice to generate *Runx2*^fl/+Cre Rosa tomato^ mice and *Runx2*^fl/flCre Rosa tomato^ mice. We examined the percentages of the osteoblasts derived from hypertrophic chondrocytes in total osteoblasts (tomato^+^mTFP^+^/tomato^+^). About 30%, 20%, and 15% of osteoblasts in the trabecular bone was derived from chondrocytes in control *Runx2*^fl/+Cre Rosa tomato^ mice at E17.5, newborn, and 1 week of age, respectively, whereas they were 1-3% in *Runx2*^fl/flCre Rosa tomato^ mice (Fig. 6). In the endosteum of cortical bone at 1 week of age, about 4% of osteoblasts was derived from chondrocytes in *Runx2*^fl/+Cre Rosa tomato^ mice and they were absent in *Runx2*^fl/flCre Rosa tomato^ mice (Fig. 7A-G). At 3 weeks of age, about 15% of osteoblasts in the trabecular bone and in the endosteum of cortical bone in *Runx2*^fl/+Cre Rosa tomato^ mice was derived from chondrocytes (Fig. 7H-Q). These were described in the Result section (pages 16-17).

microCT analysis shows that long bones and vertebra have normal properties at 6 and 20 weeks of age. It may be worth showing that the strength and material properties of these bones are also normal at these time points.

We performed three-point bending test at 12 weeks of age. The maximum load, displacement, stiffness, and energy to failure were similar between *Runx2*^fl/fl^ and *Runx2*^fl/flCre^ mice, showing that bone strength of *Runx2*^fl/flCre^ mice was normal (Fig. 13). These were described in the Result section (page 20).

Minor comments
Skeletal preps in figure 1 should show high magnification of selected skeletal elements.

We showed the hind limbs of skeletal preparation at a higher magnification (Fig. 1D, E).

Reviewer #2: In this study, Qin and colleagues report that conditional loss of Runx2 in hypertrophic chondrocytes does not affect the formation of the bone collar and blood vessel invasion of the developing growth plate but impairs the transdifferentiation of hypertrophic chondrocytes into osteoblasts at the primary spongiosa site. Notably, however, bone mass accrual in both the trabecular and cortical compartments of young adult mice appeared to be normal as shown by microCT analysis.

The paper is interesting, but numerous issues need to be addressed to strengthen the authors’ conclusions and their biological significance.

Specific comments:
1. The Title is confusing and misleading.

We changed the title to “Runx2 is essential for the transdifferentiation of chondrocytes into osteoblasts”.

2. The Text needs careful editing. In particular, the use of “..under the growth plate..” terminology is unclear. The authors may consider replacing it with “..at the border between the growth plate and primary spongiosa..” or similar sentence.

We changed the description to “in the region of presumptive primary spongiosa”, or “in the region of primary spongiosa” according to the developmental stage.

3. Findings in Runx2fl/+Cre mice need to be shown to exclude the possibility that some aspects of the phenotype could be secondary to the expression of Cre recombinase per se.

We added the data of *Runx2*^fl/+Cre^ mice at E16.5 in Fig. 2. *Runx2*^fl/fl^ mice and *Runx2*^fl/+Cre^ mice were similar.

4. The authors need to specify what bones have been analyzed throughout the Text.

We described the names of bones that were used in the experiments in the text.

5. Figure 3 panels Q-V: It is not clear where TUNEL positive cells have been quantified, growth plate, or bone marrow? Please, specify.

TUNEL-positive cells were counted in the regions that correspond to the boxed regions in P and Q, which are magnified in P’ and Q’, using the serial sections. This is described in the legend of Fig. 3 (page 45).

6. Figure 4: VEGF IHC is not very convincing. It would be helpful to show the negative controls. In addition, VEGF mRNA should also be investigated to complement the IHC findings. Lastly, the number of blood vessels at the border between the growth plate and primary spongiosa should be quantified.

We included the negative controls of immunohistochemistry using anti-Vegfa antibody in Fig. 4.

Vegfa mRNA was quantified by real-time RT-PCR using the RNA extracted from the hypertrophic and terminal hypertrophic layers at E15.5, which were isolated by laser capture microdissection system. Vegfa mRNA in *Runx2*^fl/flCre^ mice was significantly lower than that in *Runx2*^fl/flCre^ mice (Fig. 1Q, R).

We also performed immunohistochemistry using anti-CD34 antibody to detect endothelial cells of blood vessels at E16.5, and the invaded blood vessels were quantified. Blood vessels invaded the cartilage at a similar level in *Runx2*^fl/fl^ and *Runx2*^fl/flCre^ mice (Fig. 3I-K).

7. It would be helpful if the authors could comment on the formation of the secondary ossification center and transdifferentiation of hypertrophic chondrocytes into osteoblasts at that site.

Osteoblasts derived from chondrocytes were present in the secondary ossification center of femurs in *Runx2*^fl/+Cre LacZ^ mice but they were virtually absent in that in *Runx2*^fl/flCre LacZ^ mice at 3 weeks of age (Supplementary Figure 5A-C). We described it in the Result section (page 17).

8. More importantly, the microCT data need to be completed by static and dynamic histomorphometry analysis to strengthen the authors’ conclusion that bone mass accrual is indeed normal in young mutant mice.

Dynamic bone histomorphometry was performed in the cortical bone of femurs in 6-week-old females and 20-week-old males. MAR, MS/BS, and BFR/BS were similar between *Runx2*^fl/fl^ and *Runx2*^fl/flCre^ mice at both ages (Figs. 10 and 11).

We also performed bone histomorphometric analysis of vertebrae at 6 weeks and 12 weeks of age and tibiae at 12 weeks of age in female mice. All parameters for osteoblasts, osteoclasts, and bone resorption and formation were similar between *Runx2*^fl/fl^ and *Runx2*^fl/flCre^ mice (Fig. 12). These data were described in the Result section (page 20).

9. In principle, it would be helpful to analyze the bone phenotype in both males and females. At the minimum, the sex of the mice used for analysis should be specified.

We analyzed females at 6 weeks and 12 weeks of age and males at 20 weeks of age. We described 20-week-old mice as females in the previous submission but it was a mistake. They were males. We also specified the sex of the mice used for the analyses.

Reviewer #3: In this manuscript, authors investigated the role of Runx2 during transdifferentiation of chondrocytes into osteoblast. Conditional KO of Runx2 in hypertrophic chondrocyte impaired the transdifferentiation of chondrocytes, but did not impair the vascular invasion into cartilage. Moreover, authors also indicated the fundamental role of transdifferentiation of chondrocytes during embryonal and adult bone formation. The manuscript is well written and generally easy to comprehend. However, there are several limitations of the study. This for sure could increase the value of the manuscript.

Major comments
1. In this report, there was a change in the extent of VEGF expression, but no change in vascular invasion. However, it is difficult to see that the presented image did not change the invasion of the blood vessels. Vascular endothelium should be stained by such as PECAM1 or quantitatively evaluated. In addition, the reason why there was no difference in vascular invasion should be also discussed.

We performed immunohistochemistry using anti-CD34 antibody to detect endothelial cells of blood vessels at E16.5, and the invaded blood vessels were quantified. The blood vessels invaded the cartilage at a similar level in *Runx2*^fl/fl^ and *Runx2*^fl/flCre^ mice (Fig. 3I-K).

Vegfa conditional knockout mice using *Col2a1*-Cre, which directs the transgene expression to chondrocytes and perichondral cells, showed delayed vascular invasion into the cartilage (Zelzer et al, 2004). The delayed vascular invasion is due to the lack of Vegfa expression in terminal hypertrophic chondrocytes and perichondrial osteoblasts, both of which strongly express Vegfa. Similarly, Vegfa conditional knockout mice using *Sp7*-Cre, which directs the transgene expression to prehypertrophic chondrocytes and preosteoblasts in the perichondrium, showed delayed vascular invasion into the cartilage (Duan et al., 2015). The delayed vascular invasion is also due to the lack of Vegfa expression in terminal hypertrophic chondrocytes and perichondrial osteoblasts. Vegfa expression in the terminal hypertrophic chondrocytes was reduced in *Runx2*^–/–^ mice and *Runx2*^fl/flCre^ mice, but vascular invasion into the cartilage never occurs in *Runx2*^–/–^ mice but normally occurred in *Runx2*^fl/flCre^ mice. The difference of the two mouse models is the presence of perichondrial osteoblasts in *Runx2*^fl/flCre^ mice but their absence in *Runx2*^–/–^ mice. Thus, these findings indicate that Vegfa expression in perichondrial osteoblasts is sufficient for the vascular invasion into the cartilage. These discussion was described in the Discussion section (pages 23-24).

2. In Runx2fl/fl cre mice, the apoptosis of hypertrophic chondrocyte was enhanced, however apoptosis itself is a phenomenon normally observed in bone differentiation. It is necessary to show or discuss the mechanism. The changes of known factors involved in chondrocyte apoptosis such as Bcl-2 (Oshima et al., JBC 2008.) might be helpful.

We compared the expression of the genes in hypertrophic and terminal hypertrophic layers between *Runx2*^fl/fl^ and *Runx2*^fl/flCre^ mice at E15.5 using laser capture microdissection system (Fig. 1Q). The expression of *Bcl2*, *Pten*, *Fas*, and *Bnip3* in *Runx2*^fl/flCre^ mice was higher than that in *Runx2*^fl/fl^ mice, suggesting that Pten, Fas, and Bnip3 may be involved in the increased apoptosis in *Runx2*^fl/flCre^ mice (Fig. 1R). These were described in the Result section (Page 10).

3. Osteoclast increased in Runx2fl/fl cre mice. Number of osteoclasts might have strong impact on bone formation and remodeling. The authors discussed that necrosis had increased DAMPs and induced osteoclastgenesis. How did the increase of osteoclast have an effect on bone formation in this model? This issue should be discuss.

The increase of osteoclasts may have promoted the invasion of blood vessels and perichondrial preosteoblasts into the cartilage by removing the cartilaginous matrix. It was discussed in the Discussion section (Pages 24-25).

Minor comments
1. Also comment or discuss the bones other than the femur in Runx2fl/fl cre mice.

We also analyzed vertebrae and tibiae by micro-CT and/or bone histomorphometric analysis. They were also normal in *Runx2*^fl/flCre^ mice (Figs. 10-12).

2. In Fig 5, Runx2fl/fl cre and Runx2fl/+ cre were compared. Please show or comment about the differences of the phenotype in Runx2fl/+ cre and Runx2fl/+ or wild type.

We added the data of *Runx2*^fl/+Cre^ mice at E16.5 in Fig. 2. There was no difference between *Runx2*^fl/fl^ and *Runx2*^fl/+Cre^ mice.

3. The numbers in the figures are included in the histological images and are difficult to see. Make it easier to see as much as possible.

We put the numbers outside of the figures.
